# Geminivirus-derived replicons: assessment for transient GFP expression in tobacco and tomato

**DOI:** 10.3389/fpls.2026.1857507

**Published:** 2026-06-03

**Authors:** Nikolaos Tsakirpaloglou, Ourania Melita, Athanasios Kaldis, Alexios Polidoros, Andreas Voloudakis

**Affiliations:** 1 Laboratory of Plant Breeding and Biometry, Faculty of Crop Science, Agricultural University of Athens, Athens, Greece; 2 Laboratory of Genetics and Plant Breeding, Faculty of Agriculture, Aristotle University of Thessaloniki, Thessaloniki, Greece

**Keywords:** geminivirus-derived replicons (GVRs), genome editing, green fluorescence protein (GFP), molecular farming, *Nicotiana tabacum*, *Solanum lycopersicum*

## Abstract

Global food security requires innovative strategies for sustainable crop improvement. Gene editing offers a precise and rapid approach to plant modification, but its success depends on efficient delivery and robust expression systems. Geminivirus-derived replicons (GVRs) enhance transient expression by amplifying introduced DNA within plant cells. In this study, we evaluated three previously deconstructed geminiviral backbones -Bean yellow dwarf virus (BeYDV), Tomato leaf curl virus (ToLCV), and Wheat dwarf virus (WDV)- against a non-replicating T-DNA control for their ability to sustain GFP expression in tobacco (*Nicotiana tabacum*) and tomato (*Solanum lycopersicum*). Constructs were delivered via *Agrobacterium tumefaciens*, and *in planta* GVR circularization was verified, with accumulation levels dependent on the specific replicon and host species. GFP RNA and protein accumulation was assessed by RT–qPCR, fluorescence imaging, and ELISA; all GVRs prolonged GFP fluorescence relative to control. In tobacco, transcript levels increased significantly by 3 days post infiltration (dpi), reaching up to 221- fold by 6 dpi with BeYDV, while BeYDV and ToLCV produced approximately fivefold higher protein levels. In tomato, ToLCV and WDV showed the strongest enhancement, with transcript and protein levels increasing up to 6.3-fold and 2.4-fold, respectively. These results demonstrate that GVRs markedly enhance and extend transient gene expression in solanaceous hosts, with performance dependent on the replicon and plant species. ToLCV and BeYDV were most effective in tobacco, whereas ToLCV and WDV performed best in tomato. Overall, GVRs represent versatile tools for transient protein production and for improving the delivery and efficiency of genome-editing reagents in plants.

## 1 Introduction

Ensuring an adequate food supply has been a continuous challenge throughout human history. Today, global food security is increasingly threatened by population growth, resource limitations, and environmental as well as geopolitical instability. Factors such as climate change effects, armed conflicts, consumer preferences changes, and global health crises further increase the vulnerability of food systems. With the world population projected to surpass 9.7 billion by 2050 (Çakmakçı et al., 2024), the demand for safe, nutritious, and sustainable food will require innovative strategies in resource management and agricultural production. A key requirement for achieving global sustainability is the improvement of crop yield, resilience, and nutritional quality (Food and Agriculture Organization of the United Nations FAO, 2018; Smol, 2023). While traditional breeding and selection have historically driven major advances in agriculture, these methods are often slow, labor-intensive, and limited by the available genetic diversity. To meet future demands, innovative approaches are needed that allow for faster, more precise, and predictable crop improvement (Bradbury et al., 2025). Recent breakthroughs in gene editing have provided powerful tools to accelerate plant breeding by reducing the breeding cycle time, addressing the global food needs within much shorter timeframes than classical plant breeding (Chen et al., 2019; Gao, 2021). However, several technical and biological challenges remain before the full potential of large-scale plant gene editing can be realized (de Lange et al., 2022; Nasti and Voytas, 2021).

The use of viruses as vehicles for delivering genome editing components has been extensively explored lately in both plant and mammalian systems (Wang et al., 2019; Asmamaw Mengstie, 2022; Prestwich et al., 2024; Mahmood et al., 2025). In plants, viral vectors offer a promising alternative to conventional approaches such as generating stably transformed plants or using transiently modified plant tissues (Mach, 2014; Mahmood et al., 2025). Within this context, geminiviruses, a large family of plant single-stranded DNA viruses that infect a wide range of plant species, have been engineered for protein expression using two main strategies: the full-virus and deconstructed-virus approaches (Gleba et al., 2004) or as tools for functional genomics (Bhattacharjee and Hallan, 2022). In the “full-virus” strategy, geminiviruses retain most or all of the genetic elements necessary for replication and systemic spread within the host. Heterologous sequences can be introduced into the viral genome by replacing the coat protein gene, which is dispensable for cell-to-cell movement in some bipartite begomoviruses (Gardiner et al., 1988). Using this approach, Tomato golden mosaic virus and African cassava mosaic virus were successfully modified to express neomycin phosphotransferase (Hayes et al., 1988) and chloramphenicol acetyltransferase (Ward et al., 1988), respectively, in tobacco plants.

To improve the efficiency of plant genome editing, Baltes et al. (2014) introduced a simplified geminivirus-based vector system using a “deconstructed virus” strategy. This approach retained only the essential replication elements of the virus, such as replication-related proteins, the large and short intergenic regions, and the conserved nucleotide sequence (TAATATTAC), while removing non-essential genes, including those encoding movement and coat proteins, to bypass genome size constraints. Delivery of these geminivirus-derived replicons (GVRs) is achieved through *Agrobacterium*-mediated infiltration, particle bombardment, or other transient transformation methods. Using this system, Baltes et al. demonstrated that Bean yellow dwarf virus (BeYDV)-based GVR could efficiently deliver sequence-specific nucleases (ZFNs, TALENs, and CRISPR-associated nucleases) along with repair templates in *Nicotiana tabacum*, achieving high-frequency targeted mutagenesis and precise correction of a disrupted GUS: NPTII transgene (Baltes et al., 2014). Similarly, BeYDV- and Tomato leaf curl virus (ToLCV)-based replicons enabled high-efficiency targeted insertions in the tomato genome (Čermák et al., 2015), underscoring the potential of GVRs to overcome the long-standing efficiency barrier in plant gene targeting.

Since these initial studies, GVRs have been successfully applied to a wide range of plant species and target genes. In potato, they facilitated CRISPR/Cas-mediated introduction of herbicide-resistance edits into the *ALS1* gene (Butler et al., 2016), while in *Arabidopsis*, they enabled ZFN-, TALEN-, and CRISPR/Cas9-mediated gene insertion, though with markedly higher efficiencies in somatic than in germinal tissues (Shan et al., 2018). BeYDV-based GVRs have also achieved efficient homology-directed repair (HDR) in rice, generating pre-harvest sprouting-resistant lines via *OsERF1* editing and introducing SNPs into *LcyE* with up to 90% editing efficiency (Kim et al., 2022, 2024a). Similarly, BeYDV-based GVRs enhanced genome editing in apple (Negishi et al., 2024), cotton (Li et al., 2022), and tomato (Dahan-Meir et al., 2018), and have supported the evaluation of novel compact nucleases such as CasΦ (Cas12j) and Cas12f variants (Gong et al., 2024). Expanding their application to cereals, a deconstructed Wheat dwarf virus (WDV) replicon system drove more than 100-fold increases in transgene expression and enabled multiplexed and simultaneous HDR-based gene targeting across all three homeoalleles of a hexaploid wheat at frequencies of approximately 1% (Gil-Humanes et al., 2017). Furthermore, GVRs carrying CRISPR/LbCpf1 components significantly enhanced HDR efficiency in tomato, achieving a threefold increase with a *de novo* multi-replicon system. It was observed that physical conditions, such as temperature and light, substantially influence editing outcomes (Vu et al., 2020). Additional GVRs have also proven effective: for example, an episomal replicon derived from Croton yellow vein mosaic virus (CYVMV) supported GFP expression in tobacco (Jailani et al., 2016), while a Sweet potato leaf curl virus-based GVR enabled robust *Agrobacterium*-mediated transient expression in duckweed species (Peterson et al., 2021). Collectively, these studies illustrate that GVRs significantly enhance the efficiency of targeted genome modification by providing high local copy numbers of both genome editing nucleases and donor templates, enabling precise sequence modification across diverse plant species.

Other viral-replicon-based platforms have been developed to enhance the delivery and expression of genome editing reagents and transgenes in plants. Tobacco mosaic virus (TMV)-derived TRBO vectors have been used to transiently deliver sgRNAs, achieving up to 70% indel frequencies in *Nicotiana benthamiana* within seven days, despite retaining 5′ sgRNA overhangs, suggesting a novel *in planta* processing mechanism (Cody et al., 2017). Similarly, Potato virus X (PVX)-based vectors have enabled efficient multiplex editing in adult solanaceous plants by expressing arrays of unspaced sgRNAs, generating approximately 80% biallelic mutations and heritable, virus-free edited progeny (Uranga et al., 2021). Barley stripe mosaic virus (BSMV)-mediated sgRNA delivery, in Cas9-expressing wheat, enabled multiplex editing and heritable promoter deletions of agronomic genes (Wang et al., 2022).

In addition, plants offer several advantages as biofactories (molecular pharming) for recombinant protein production, including low production costs, scalability, and safety from human pathogens (Liu and Timko, 2022). However, despite numerous advances, only a limited number of plant-produced pharmaceuticals have reached commercial use, largely due to low yields and inconsistent protein quality caused by fundamental differences between plant and animal expression systems. To address these limitations, GVRs have been developed to boost copy number and expression levels of biological components. For example, a BeYDV-based system enabled ethanol-inducible Rep expression to trigger episomal replicon release and amplification from stably integrated T-DNA, increasing transgene mRNA levels up to 80-fold and protein accumulation up to 10-fold in *N. tabacum* cell cultures and potato plants (Zhang and Mason, 2006). Similarly, deconstructed GVRs derived from Tomato yellow leaf curl virus (TYLCV), Honeysuckle yellow vein virus (HYVV), and Beet mild curly top virus (BMCTV) achieved rapid and robust recombinant protein production in *N. benthamiana* leaves within three days (Kim et al., 2024b).

In the present study, we evaluated the performance of the simplified GVR systems (Baltes et al., 2014) for sustaining transient expression of green fluorescent protein (GFP) in *N. tabacum* (tobacco) and *Solanum lycopersicum* (tomato). We confirmed GVR circularization in both species, monitored GFP accumulation at both transcript and protein levels over time and compared the different GVRs (BeYDV, ToLCV, and WDV) to determine which most effectively supports prolonged GFP expression. These findings provide insights into optimizing GVRs for transient gene expression and demonstrate their potential to enhance the efficiency of transiently delivered genome editing components and recombinant protein production in these species.

## 2 Materials and methods

### 2.1 Plant growth and experimental design

Tobacco (*Nicotiana tabacum* cv. Xanthi) and tomato (*Solanum lycopersicum* cv. Micro-Tom) were cultivated in a controlled-environment growth chamber under a 16 h light/8 h dark photoperiod with light intensity 250 μmol.m^-2^.s^-1^, with day/night temperatures of 25 °C/22 °C. Plants were grown individually in 270 mL pots containing nutrient-enriched black peat and perlite, arranged in trays according to a completely randomized block design. Each treatment was conducted with three biological replicates, each represented by a single plant. In tobacco, two leaves per plant were agroinfiltrated at each time point (3 and 6 days post infiltration [dpi]), whereas in tomato, two cotyledons per plant were infiltrated at 4 and 7 dpi. Plants infiltrated with untransformed *Agrobacterium tumefaciens* served as negative controls. Independent experimental runs were performed for genomic DNA isolation, fluorescence imaging, RNA extraction, and protein analysis.

### 2.2 Plasmid preparation and agroinfiltration

Four binary reporter plasmids were generated to evaluate GFP expression in tobacco and tomato. Each construct carried the prCmYLCV::GFP cassette (pMOD_C3003; Addgene #91095, Watertown, MA, USA) assembled by Golden Gate cloning (Čermák et al., 2017) and inserted into pTRANS backbones lacking a replicon (pTRANS-220; Addgene #91113) or containing BeYDV (pTRANS-221; Addgene #91115), ToLCV (pTRANS-222; Addgene #91116), or WDV (pTRANS-223; Addgene #91117) replicons. Filler modules pMOD_A0000 (Addgene #90997) and pMOD_B0000 (Addgene #91058) were used to complete modules A and B. Constructs were screened by colony PCR and verified by whole-plasmid sequencing (Oxford Nanopore; Eurofins Genomics GmbH, Germany).

Binary plasmids carrying the GFP expression cassette were introduced into *Agrobacterium tumefaciens* GV3101 by the freeze–thaw method (Weigel and Glazebrook, 2006). Cultures were resuspended in infiltration buffer (10 mM MES, 10 mM MgCl_2_, 200 µM acetosyringone) and adjusted to OD_600_=0.6. In tobacco, two leaves of 27-day-old (from seed planting) *N. tabacum* plants were infiltrated per plant, with ~1 mL bacterial suspension per leaf. In tomato, cotyledons of 15-day-old (from seed planting) *S. lycopersicum* seedlings were infiltrated. Plants were maintained under standard growth conditions after infiltration.

### 2.3 Genomic DNA isolation and PCR

Genomic DNA was extracted from infiltrated tobacco leaves and tomato cotyledons as previously described (Allen et al., 2006). Three biological replicates were performed. Replicon circularization was assessed by PCR using primers NT-83-prCmYLCV-R1 (5′-TAAAGGCAGCCGACCTAACC-3′) and NT-86-PeaE9-UTR-F2 (5′-TGTGTCAAATCGTGGCCTC-3′) with KAPA Taq PCR Kit (Roche Molecular Systems, USA) under the following conditions: 95 °C for 3 min; 26 cycles (tobacco) or 35 cycles (tomato) of 95 °C for 30 s, 60 °C for 30 s, and 72 °C for 90 s; and a final extension at 72 °C for 3 min. The *F-box* gene (Liu et al., 2012) and *EF-1* gene (Choi et al., 2018; primers Solyc-EF-1-789F: 5′-GATTGGTGGTATTGGAACTGTC-3′ and Solyc-EF-1-918R: 5′-AGCTTCGTGGTGCATCTC-3′) were used as internal controls for tobacco and tomato, respectively. Amplicon identity was confirmed by Oxford Nanopore sequencing (Eurofins Genomics GmbH, Germany).

### 2.4 Leaf fluorescence determination via ImageJ analysis

GFP fluorescence in infiltrated areas of tobacco leaves and tomato cotyledons was quantified from images acquired under blue/green LED illumination (470–520 nm) at 3 and 6 dpi (tobacco) and 4 and 7 dpi (tomato) using a FASTGene FAS-DIGI-PRO system (NIPPON Genetics EUROPE, Germany). Images were analyzed in ImageJ (Schneider et al., 2012) after separation into red, green, and blue channels. Fluorescence was measured in 4 randomly selected regions per biological replicate in the green and red channels. GFP intensity (green) was normalized to chlorophyll autofluorescence (red) to obtain a GFP/Chl ratio. A total of four measurements from three biological replicates (individual plants) per treatment and per time point were analyzed in tobacco and tomato. Plants infiltrated with untransformed (empty) agrobacteria were used as negative control.

### 2.5 RNA Isolation, cDNA synthesis, and reverse-transcription quantitative PCR

For tobacco, two infiltrated leaves from three individual plants per treatment were collected separately at 3 and 6 dpi; for tomato, two infiltrated cotyledons from three individual plants per treatment were collected separately at 4 and 7 dpi; all samples were immediately frozen in liquid nitrogen. Total RNA was extracted using TRI Reagent^®^ (Molecular Research Center Inc., USA) following the manufacturer’s instructions, and quantified spectrophotometrically (Multiskan FC, Thermo Fisher Scientific, USA). To remove genomic DNA, equal amounts of RNA were treated with TURBO DNase (Thermo Fisher Scientific, USA), followed by phenol extraction and ethanol precipitation. RNA was resuspended in UltraPure™ DNase/RNase-free distilled water (Thermo Scientific, USA).

Complementary DNA (cDNA) was synthesized from 200 ng of total RNA per sample in a 10 µL reaction volume using the FIREScript cDNA synthesis kit (Solis BioDyne, Estonia). Both oligo-dT and random primers were included to ensure comprehensive cDNA synthesis. The reverse transcription reaction was performed in a LabCycler 48S Gradient (SENSOQUEST, Germany) with the following thermal cycling conditions: 10 min at 27 °C, 60 min at 37 °C, and 5 min at 85 °C.

Reverse Transcription Quantitative PCR (RT-qPCR) was performed on a StepOnePlus™ Real-Time PCR System (Thermo Fisher Scientific, USA) using 5× HOT FIREPol EvaGreen qPCR Supermix (Solis BioDyne, Estonia). *GFP* transcript levels were amplified using the primers GFP_NT_F2 (5′-CAGTGCTTCTCCCGTTACCC-3′) and GFP_NT_R1 (5′-TGCCGTTCTTTTGCTTGTCG-3′) in triplicate under the following cycling conditions: initial denaturation at 95 °C for 12 min, followed by 40 cycles of 95 °C for 20 s, 58 °C for 20 s, and 72 °C for 60 s. The *F-BOX* gene (Liu et al., 2012) and the *TIP41* gene (Choi et al., 2018) were used as internal reference controls for tobacco and tomato samples, respectively. Relative GFP expression levels were calculated using the 2^−ΔΔCt^ method (Schmittgen and Livak, 2008).

### 2.6 Protein extraction and quantification with ELISA

For protein extraction, two infiltrated leaves from three individual tobacco plants per treatment were collected separately at 3 and 6 dpi; for tomato, two infiltrated cotyledons from three individual plants per treatment were collected separately at 4 and 7 dpi; all samples were pulverized in liquid nitrogen. Proteins were extracted using a trichloroacetic acid (TCA)/acetone precipitation method adapted from Isaacson et al. (2006). Briefly, the ground tissue was incubated overnight at −20 °C in 10% (v/v) TCA in acetone, and the resulting protein pellets were washed repeatedly with ice-cold acetone. Pellets were then resolubilized in 500 µL of 50 mM Tris-HCl by thorough vortexing. Protein concentrations were determined using the Bradford assay (Serva, Cat. No. 39222.01), yielding average concentrations of approximately 200 ng/µL for tobacco and 20 ng/µL for tomato samples.

For GFP quantification, protein extracts were analyzed using the PathScan Total GFP Sandwich ELISA Kit (Catalog No. 7878, Cell Signaling Technology), following the manufacturer’s instructions. Before the assay, samples were diluted 1:1,000 (tobacco) or 1:20 (tomato) in the provided sample diluent. According to the standard protocol, samples were incubated with the color-developing reagent for 10 min at 37 °C. Reactions were stopped with stop solution, and absorbance was measured at 450 nm using a Multiskan FC Photometer (Thermo Fisher Scientific, USA).

### 2.7 Statistical analysis

Statistical analyses of leaf fluorescence, relative gene expression, and protein levels were performed to compare differences among plant groups. One-way ANOVA was used to evaluate treatment effects, and significant differences (*p* < 0.05) were further assessed using Tukey’s Honestly Significant Difference (HSD) *post hoc* test. Pairwise comparisons between the two time points, within the same treatment, were conducted using a two-tailed, homoscedastic Student’s t-test. In all analyses, a *p*-value less than 0.05 was considered statistically significant.

## 3 Results

### 3.1 Vector design for GFP expression in plants

**Figure 1:**
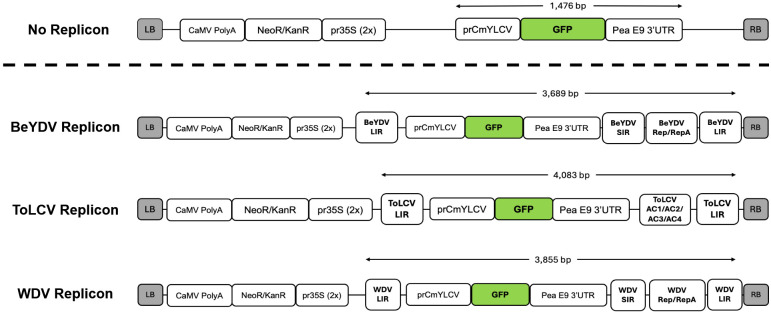
Schematic representation of the genetic cassettes of the GVRs and the “No Replicon” control used. All constructs are shown with their respective T-DNA sizes. Key elements include the left (LB) and right (RB) borders; the *GFP* expression cassette driven by the CmYLCV promoter and terminated by the pea rbcS E9 3′UTR; and the kanamycin resistance marker (NeoR/KanR) under the enhanced CaMV 35S promoter (pr35S 2×) with the CaMV polyA signal. Replication elements were derived from Bean yellow dwarf virus (BeYDV), Tomato leaf curl virus (ToLCV), or Wheat dwarf virus (WDV), consisting of long intergenic repeats (LIR), short intergenic repeats (SIR), and the corresponding replication-associated proteins (Rep/RepA for BeYDV and WDV; AC1–AC4 for ToLCV).

To assess the effect of GVR sequences on transient expression, four binary GFP expression vectors were generated (**Figure 1**). All constructs included the essential elements for *Agrobacterium*-mediated delivery and T-DNA expression, with *GFP* driven by the CmYLCV promoter. The “No Replicon” control vector (Construct 1, T-DNA size: 4,088 bp) lacked viral sequences, while BeYDV GVR (Construct 2, T-DNA size: 5,907 bp), ToLCV GVR (Construct 3, T-DNA size: 6,326 bp), and WDV GVR (Construct 4, T-DNA size: 6,065 bp) contained replication elements, such as long intergenic repeats (LIRs), short intergenic repeats (SIRs), and replication-associated proteins from Bean yellow dwarf virus (GenBank DQ458791.1), Tomato leaf curl virus (GenBank DQ629101.2), and Wheat dwarf virus (GenBank MK193742.1), respectively. These vectors enabled a direct comparison of GVRs in terms of GFP expression efficiency, stability, and host interactions.

Sequence alignment of the respective long and short intergenic regions, as well as the associated replication proteins, revealed low sequence similarities among the three GVRs (**Supplementary Figures 1**, **2**). In the mastreviruses BeYDV and WDV, two ORFs encoding the replication-associated proteins, Rep and RepA, were incorporated into the GVR backbones. Rep serves as the key initiator of geminiviral replication, binding to a conserved nonanucleotide sequence (TAATATTAC) located at the apex of a stem-loop within the origin of replication, where it introduces a site-specific nick to initiate the rolling-circle replication (Bhattacharjee and Hallan, 2022). In contrast, the bipartite begomovirus ToLCV GVR included four replication-associated ORFs (AC1-AC4) in its deconstructed configuration, including the silencing suppressor (AC2 and AC4) and the transcriptional activator (AC2) proteins.

### 3.2 *In planta* circularization of the GVRs

**Figure 2:**
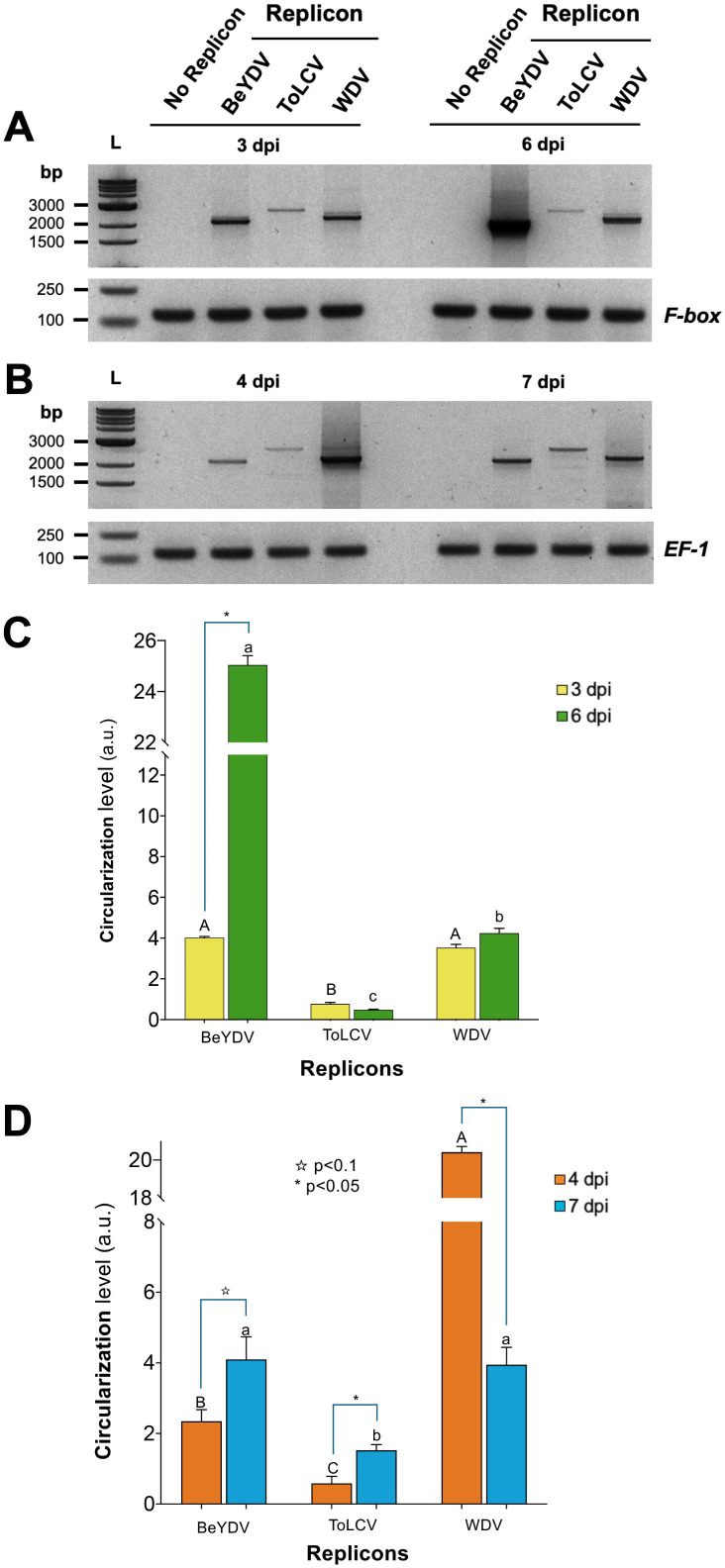
PCR on genomic DNA to verify replicon circularization at different time points after inoculation in **(A)** tobacco leaves and **(B)** tomato cotyledons. *F-box* and *EF-1* primers were used as species-specific controls. L: The FastGene 1 kb (100 bp - 10,000 bp) DNA marker Plus (NIPPON Genetics EUROPE, DE). Circularization levels of GVRs quantified by ImageJ in **(C)** tobacco leaves at 3 and 6 dpi and **(D)** tomato cotyledons at 4 and 7 dpi following infiltration with plasmids carrying BeYDV, ToLCV, or WDV GVRs. Values represent means ± SE from three measurements obtained from three infiltrated leaves or cotyledons per treatment. Different uppercase and lowercase letters indicate significant differences among treatments within the same time point (p < 0.05). Asterisks (*, ✡) indicate significant differences between time points within the same treatment (p < 0.05 and p < 0.1, respectively). a.u., arbitrary units.

To verify *in planta* GVR circularization, genomic DNA was isolated from agroinfiltrated tobacco leaves and tomato cotyledons at two time points and analyzed by PCR (**Figures 2A, B**) using primers, for all replicons, that target the CmYLCV promoter (NT-083-R1) and the PeaE9-UTR (NT-086-F2) elements of the GFP cassette. Amplification and sequence analysis confirmed successful (and potentially differential) circularization of the BeYDV, ToLCV, and WDV replicons in both tobacco and tomato tissues, which was maintained over time in both species. Despite the inherent limitations of PCR-based comparisons (e.g., amplification kinetics, amplicon size, and GC content, etc.), the mastrevirus-derived BeYDV and WDV replicons appeared to circularize more efficiently than for the begomovirus-derived ToLCV in both tobacco and tomato. In tobacco, circularization values at 3 dpi were 4 and 3.5 for BeYDV and WDV, respectively, compared with 0.8 for ToLCV, while replicon levels at 6 dpi reached 25 and 4.2, respectively, compared with 0.5 for ToLCV (**Figure 2C**). In tomato, corresponding values at 4 dpi were 2.3 and 20.4 for BeYDV and WDV, respectively, compared with 0.6 for ToLCV, whereas at 7 dpi the values were 4 and 3.9, respectively, compared with 1.5 for ToLCV (**Figure 2D**). ToLCV circularization decreased over time in tobacco but increased in tomato. BeYDV showed the highest circularization efficiency in tobacco, with a pronounced increase in replicon accumulation at 6 dpi, whereas WDV was the predominant replicon in tomato, reaching peak levels at 4 dpi.

### 3.3 Time-course analysis of GFP expression following agroinfiltration

**Figure 3:**
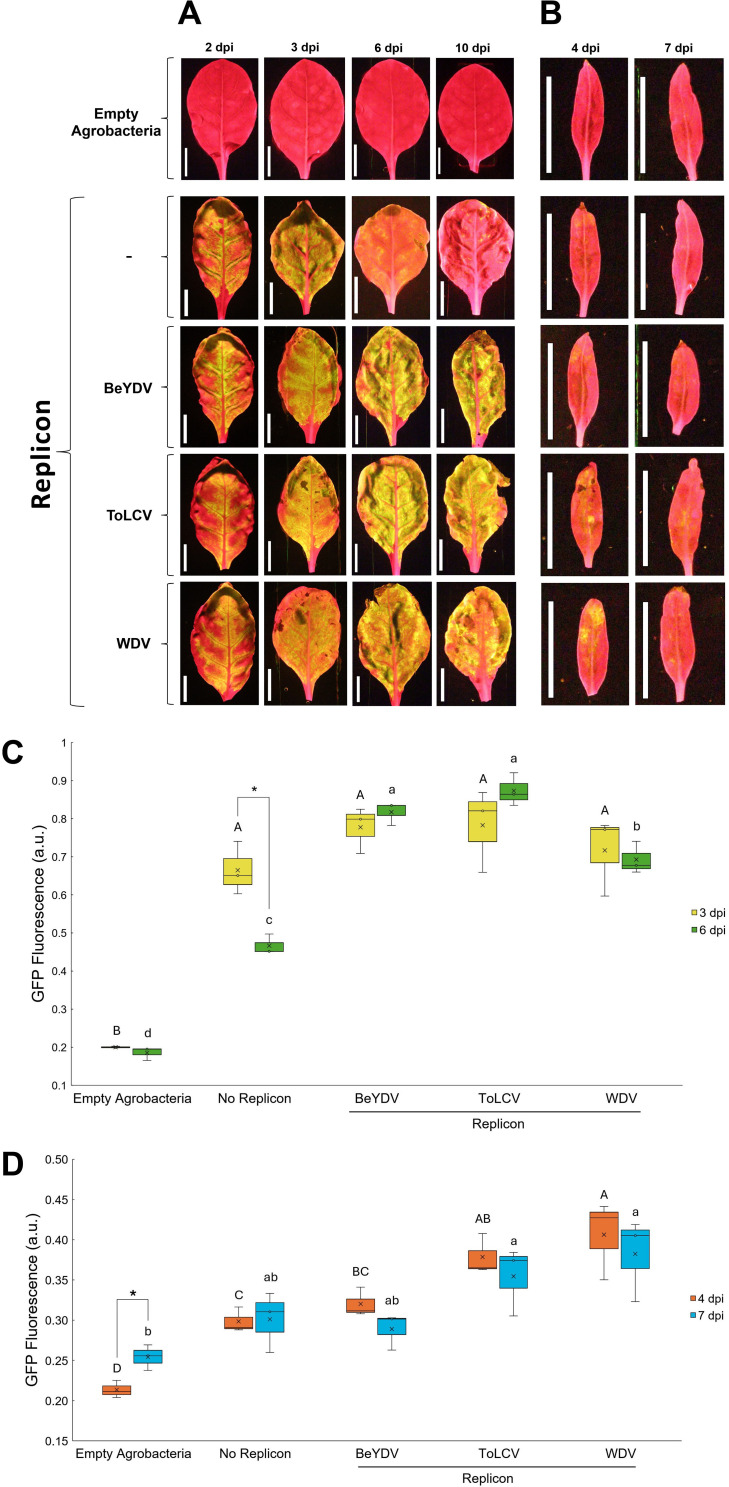
Time-course of GFP expression following agroinfiltration in **(A)** tobacco leaves (2, 3, 6, 10 dpi) and **(B)** tomato cotyledons (4, 7 dpi), illustrating transient expression dynamics after delivery of GVR plasmids. Fluorescence images were captured under blue/green LED illumination (470–520 nm); scale bar = 1.5 cm. Quantification of GFP fluorescence by ImageJ in **(C)** tobacco leaves (3, 6 dpi) and **(D)** tomato cotyledons (4, 7 dpi) infiltrated with plasmids lacking a replicon or carrying BeYDV, ToLCV, or WDV GVRs. Values represent means ± SE (n = 4 measurements from three infiltrated leaves or cotyledons per treatment). Capital and small letters denote significant differences among treatments at the same time point (p < 0.05), and the asterisk (*) indicates significant differences between time points within treatments (p < 0.05). a.u.: arbitrary units.

To assess potential differences in foreign gene expression due to the GVR sequences, a time-course analysis of GFP expression in tobacco leaves and tomato cotyledons was performed following agroinfiltration with the different GVR constructs (**Figure 3**). GFP fluorescence was monitored under Blue/Green LED illumination at 2, 3, 6, and 10 dpi for tobacco and at 4 and 7 dpi for tomato.

As expected, for both tobacco and tomato, the negative control plants (infiltrated with untransformed agrobacteria) showed no detectable GFP signal throughout the time course, displaying only the red autofluorescence characteristic of chlorophyll.

In particular, for tobacco (**Figure 3A**), all other treatments produced detectable GFP fluorescence, which varied in intensity and persistence. The “No Replicon” construct, which expresses GFP without a GVR, exhibited moderate fluorescence at 2 and 3 dpi. However, signal intensity decreased substantially by 6 dpi and was nearly undetectable by 10 dpi, indicating limited transient expression over time. Conversely, constructs containing GVRs (BeYDV and ToLCV), produced strong GFP fluorescence as early as 2 dpi, which remained stable through 10 dpi indicating efficient replication and prolonged gene expression. However, the construct containing the WDV GVR showed declining GFP fluorescence over time (this being clearer at 10 dpi). These results indicate that incorporating GVRs (most notably BeYDV and ToLCV) significantly enhances both the intensity and duration of GFP expression in tobacco leaves, compared to the non-replicating construct.

In tomato cotyledons (**Figure 3B**), the “No Replicon” construct exhibited weak GFP fluorescence at 4 dpi, which declined markedly by 7 dpi, indicating poor transient expression over time. In contrast, all GVR constructs (BeYDV, ToLCV, and WDV) produced markedly stronger fluorescence at 4 dpi, with ToLCV and WDV showing the highest initial signal intensity. Although fluorescence from the GVR constructs gradually decreased over time, it remained consistently higher than that of the non-replicating control. Notably, ToLCV and WDV maintained elevated fluorescence at 7 dpi, suggesting more sustained gene expression compared to BeYDV. These results, consistent with observations in tobacco, indicate that GVR-based constructs significantly enhance and prolong GFP expression in tomato relative to the non-replicating construct.

GFP fluorescence in infiltrated tissues was further quantified by ImageJ (**Figures 3C, D**; **Supplementary Figure 3**). In tobacco (**Figure 3C**), fluorescence at 3 dpi was similar across treatments, but by 6 dpi the “No Replicon” control declined markedly, whereas replicon-containing samples maintained the fluorescence. ToLCV and BeYDV supported significantly higher fluorescence than WDV over time. In tomato (**Figure 3D**), ToLCV and WDV showed higher fluorescence than the control at 4 dpi, and expression was sustained over time in all treatments; however, ToLCV and WDV maintained higher levels than BeYDV and the control. Overall, these results indicate that GVRs enhanced and stabilized GFP expression relative to the “No Replicon” control, with ToLCV and BeYDV most effective in tobacco, and ToLCV and WDV in tomato.

### 3.4 *GFP* transcript quantification by RT-qPCR

**Figure 4:**
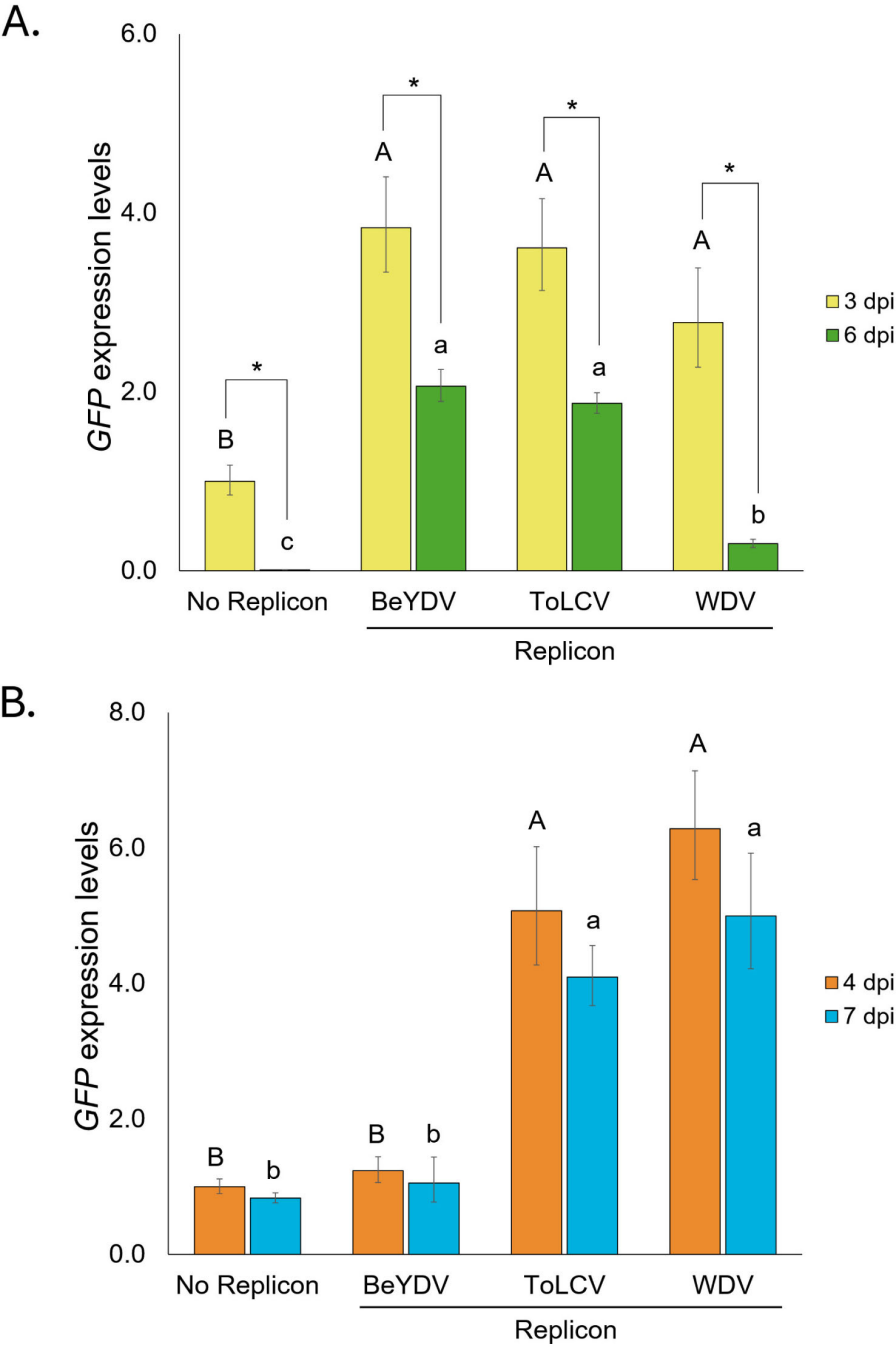
RT-qPCR analysis of GFP transcript levels in **(A)** tobacco leaves (3, 6 dpi) and **(B)** tomato cotyledons (4, 7 dpi) following agroinfiltration with plasmids lacking a replicon or carrying BeYDV, ToLCV, or WDV GVRs. Bars represent mean ± SE (n = 3 measurements from infiltrated leaves or cotyledons from three individual plants per treatment). Capital and small letters denote significant differences among treatments at the same time point (p < 0.05), and the asterisk (*) indicates significant differences between time points within each treatment (p < 0.05).

*GFP* transcript levels were quantified by RT-qPCR in agroinfiltrated tobacco leaves (3, 6 dpi) and tomato cotyledons (4, 7 dpi) using constructs lacking a replicon (“No Replicon”) or carrying BeYDV, ToLCV, or WDV GVRs (**Figure 4**). In tobacco, all GVRs increased *GFP* expression relative to the control, with 3.8-, 3.6-, and 2.8-fold increases at 3 dpi, and up to 221-, 200-, and 32-fold increases at 6 dpi for BeYDV, ToLCV, and WDV, respectively. Although transcript levels declined over time, GVR-containing constructs remained significantly higher than the control, which approached baseline by 6 dpi. In tomato, GVRs also enhanced GFP expression relative to the control, with 1.2-, 5.1-, and 6.3-fold increases at 4 dpi, and 1.3-, 5-, and 6-fold increases at 7 dpi for BeYDV, ToLCV, and WDV, respectively. Overall, GVRs enhanced and prolonged transient expression, with BeYDV and ToLCV most effective in tobacco, and ToLCV and WDV in tomato.

### 3.5 GFP protein quantification by ELISA

**Figure 5:**
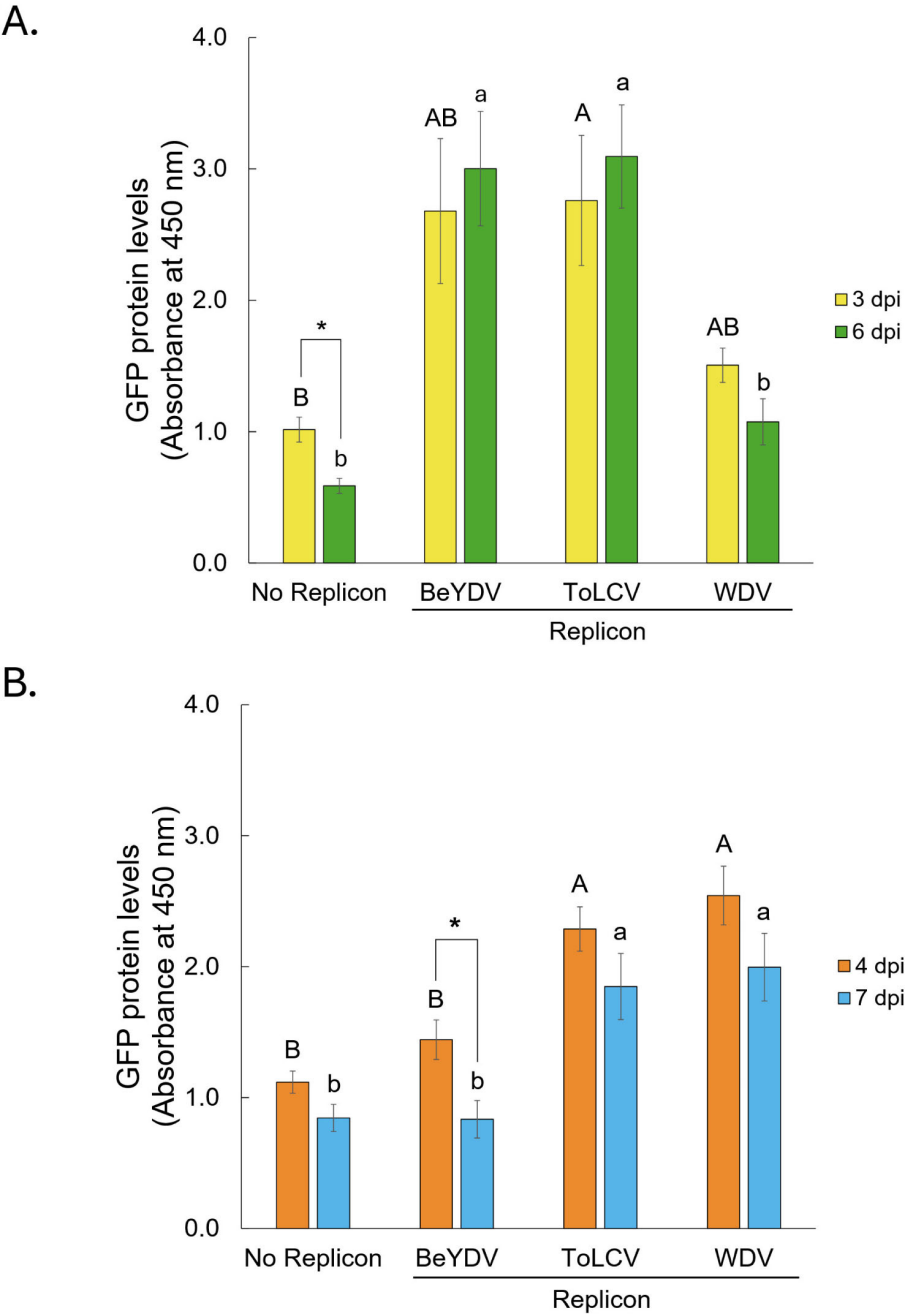
ELISA quantification of GFP protein levels in **(A)** tobacco leaves (3, 6 dpi) and **(B)** tomato cotyledons (4, 7 dpi) following agroinfiltration with plasmids lacking a replicon or carrying BeYDV, ToLCV, or WDV GVRs. Bars represent mean ± SE (n = 3 measurements from infiltrated leaves or cotyledons from three individual plants per treatment). Capital and small letters denote significant differences among treatments at the same time point (p < 0.05), and the asterisk (*) indicates significant differences between time points within each treatment (p < 0.05).

GFP protein levels were quantified by ELISA in tobacco leaves at 3 and 6 dpi and in tomato cotyledons at 4 and 7 dpi (**Figure 5**). In tobacco, all GVR constructs increased GFP accumulation at 3 dpi relative to the “No Replicon” control, with BeYDV and ToLCV showing 2.6- and 2.7-fold higher levels, respectively. By 6 dpi, GFP levels in the control declined sharply, whereas in BeYDV and ToLCV the accumulation elevated (5.1- and 5.3-fold above control). Over time, ToLCV and BeYDV sustained or increased expression, while WDV showed a moderate decline but remained above control levels.

In tomato, GFP accumulation at 4 dpi was highest using ToLCV and WDV GVRs, showing ~2.0- and 2.3-fold increases over the “No Replicon” control, consistent with transcript level differences. In contrast, BeYDV-associated GFP remained similar to the control (similar to transcript levels). By 7 dpi, ToLCV and WDV had accumulation elevated (~2.2- and 2.4-fold above control), whereas BeYDV showed no increase compared to the control. Over time, GFP levels declined significantly in BeYDV samples, while ToLCV and WDV remained stable and significantly higher than the control.

Taken together, these results demonstrate that BeYDV, ToLCV, and WDV GVRs can enhance and prolong GFP expression in solanaceous species, with ToLCV GVR showing the strongest and most sustained effect, in both tobacco and tomato. Furthermore, the BeYDV and WDV GVRs could be considered as alternatives for tobacco and tomato, respectively.

## 4 Discussion

Geminiviruses are widely used in plant biotechnology owing to their capacity for high-copy DNA replication in planta and their broad host range. These features have enabled applications in molecular production, directed evolution, and genome editing (Kim et al., 2024b; Mach, 2014; Zhu et al., 2025). Here, we evaluated a simplified GVR system, originally developed by Baltes et al. (2014), for its capacity to sustain transient GFP expression in tobacco and tomato. Deconstructed GVRs markedly enhanced both the intensity and duration of GFP expression compared with a non-replicating control. Replicons derived from BeYDV, ToLCV, and WDV increased GFP transcript and protein levels, prolonged fluorescence, and maintained detectable expression at later time points when the control had declined. These findings underscore the advantage of GVRs in amplifying delivered genetic material in planta, thereby increasing copy number and enhancing downstream RNA and protein accumulation (Baltes et al., 2014; Čermák et al., 2015).

The three GVRs displayed distinct performance profiles. ToLCV consistently supported strong and sustained GFP accumulation in both *Solanaceae* species. In contrast, BeYDV and WDV exhibited host-dependent effects: BeYDV was more effective in tobacco, whereas WDV performed better in tomato, an unexpected result given that WDV primarily infects monocot hosts (Pfrieme et al., 2023). Since potentially differential circularization was confirmed for all the GVRs in both species (**Figure 2**), these differences likely reflect variations in replicon accumulation. Previous studies verified replicon circularization for BeYDV in tobacco (Baltes et al., 2014) and for WDV in wheat (Gil-Humanes et al., 2017). Peak BeYDV accumulation has been reported at approximately 5 dpi, consistent with our observation of maximal circularization at 6 dpi in tobacco (**Figure 2A**), which may explain its superior performance relative to WDV. Conversely, the enhanced activity of WDV in tomato may be attributed to its high accumulation at 4 dpi (**Figure 2B**).

Geminiviral ssDNA is converted to dsDNA and assembled with host histones into minichromosome-like structures in the infected nuclei (Pilartz and Jeske, 1992; Ceniceros-Ojeda et al., 2016). Host chromatin assembly factors such as HIRA and FAS2 promote nucleosome deposition and can limit access to viral DNA. Very recently, it has been demonstrated that overexpression of these factors in Nicotiana benthamiana leads to the formation of repressive chromatin states linked to decreased geminivirus susceptibility (Sultana et al., 2026). In addition, plant chromatin accessibility is strongly affected by the developmental status of the tissue with young (meristematic) tissues generally maintaining a more permissive chromatin environment while differentiated tissues show more compact chromatin organization (Morao et al., 2016).

Our results suggest that deconstructed geminivirus amplification depends on the GVR used, the host species (tomato vs. tobacco), and the developmental state of the agro-infiltrated tissue (developmentally old in tomato vs. differentiated but developmentally young in tobacco). Compared with tobacco leaves, tomato cotyledons likely represent a more restrictive (Low-entropy chromatin) nuclear environment for replicon amplification, leading to a thermodynamic barrier for viral-induced cell-cycle re-entry that is necessary for geminiviral replication. Geminiviruses use host S-phase machinery for their rolling-circle replication; they induce host cell-cycle re-entry by the interaction of viral Rep/RepA proteins with the retinoblastoma-related (RBR) pathway providing a replication favorable environment for them (Zhang et al., 2025). Thus, differences in host-geminivirus interactions controlling S-phase induction may be reflected in the transient high increase in WDV GVR circularization at 4 dpi in tomato (**Figure 2**). Previous studies have reported distinct RBR-binding properties for BeYDV and WDV Rep/RepA proteins that could explain the higher accumulation of WDV replicons observed in tomato (Baltes et al., 2014; Liu et al., 1999; Collin et al., 1996).

In tomato cotyledons, restricted access to host replication factors together with rapid epigenetic silencing, including RdDM (RNA dependent DNA methylation) and repressive histone modifications, may limit the accumulation of functional viral templates. This could explain the sparse and heterogeneous GFP expression observed in tomato compared with the stronger expression in tobacco. However, because different tissues were used for agroinfiltration in the two hosts, the specific contribution of host background cannot be fully separated from tissue developmental effects. Future analyses, applying spatial cell biology *in situ* methods, including detailed chromatin segmentation and structure analysis, ChiP-seq of heterochromatin marks to reveal DNA packaging, and bisulfite PCR to enable quantitative assessment of the methylation levels of viral DNA, would help determine whether stronger epigenetic silencing on geminiviral chromatin exists in tomato cotyledon relative to tobacco leaf.

Interestingly, the ToLCV GVR showed lower accumulation than BeYDV and WDV in both *Solanaceae* species. This apparent reduction may reflect technical biases associated with PCR, including differences in amplification kinetics, GC content, and amplicon size. Notably, the ToLCV replicon (3,812 nt) is larger than BeYDV (3,337 nt) and WDV (3,446 nt). Replication efficiency has been reported to decline with increasing replicon size (Wang et al., 2017), suggesting that the larger ToLCV construct may replicate less efficiently. Alternatively, its greater length may reduce stability, making it more susceptible to degradation.

Despite its lower circularization efficiency, the ToLCV GVR consistently drove strong GFP expression in both plant species. This may reflect unique features of its genomic architecture, including the organization of LIR/SIR elements, the presence of multiple replication-associated ORFs, and enhanced compatibility with host factors in both *Solanaceae* species. Unlike the mastrevirus-derived BeYDV and WDV replicons, which encode only Rep and RepA, the ToLCV construct retains four begomovirus-specific replication-associated ORFs (AC1-AC4) (Rojas et al., 2001, 2005; Boulton, 2002; Hefferon and Dugdale, 2003; Fondong, 2013). Notably, AC2 and AC4 function as RNA silencing suppressors, counteracting host RNAi defense mechanism and promoting transcript stability and protein accumulation (Veluthambi and Sunitha, 2021; Kumar and Dasgupta, 2023). AC2 seems to be a multifunctional protein that also induces other silencing suppressors like WEL1 (Werner’s exonuclease-like 1), rgs-CaM (Regulator of gene silencing – calmodulin), RAV2 (RELATED TO ABI3 and VP1) and interacts with ADK (adenosine kinase), SAMDC1 (S-adenosyl methionine decarboxylase1) and other host proteins (Veluthambi and Sunitha, 2021). AC2, also known as the transcriptional activator protein (TrAP), may further enhance gene expression by interacting with host transcriptional machinery and potentially stimulating adjacent promoters, such as the CmYLCV promoter upstream of GFP (Hartitz et al., 1999). These functional attributes could explain the elevated transcriptional output observed for ToLCV despite its low circularization. Consistent with this interpretation, sequence comparisons revealed limited similarity among the LIR/SIR regions and replication-associated proteins of the GVRs (**Supplementary Figures 1**, **2**), suggesting that structural and functional divergence contributes to the observed differences in expression.

Host specificity and the evolutionary history of the parental viruses likely contribute to the observed differences among GVRs. WDV, a mastrevirus primarily adapted to monocot hosts (Pfrieme et al., 2023), can replicate in heterologous systems but may do so less efficiently in solanaceous dicots, as its replication proteins and iteron specificity are not fully optimized for these hosts (Lazarowitz and Shepherd, 1992). This may account for its variable performance in tobacco and tomato. In contrast, begomoviruses such as ToLCV naturally infect dicots, including tomato, and are therefore better adapted to these cellular environments (Crespo-Bellido et al., 2024), consistent with the strong expression observed in both species. Such host-range and compatibility effects have been widely reported and should be considered when selecting an appropriate GVR backbone for specific plant systems.

RT-qPCR and ELISA analyses indicate that GVRs enhance transient expression by amplifying their DNA templates, leading to increased transcript abundance (**Figure 4**) and higher protein accumulation over time (**Figure 5**). This finding aligns with previous studies showing that GVRs enhance processes such as homology-directed repair and reporter expression by amplifying repair templates and nuclease cassettes or by increasing protein yields (Chen et al., 2022; Kim et al., 2024b). The observed prolonged GFP expression in the two species further supports the role of replicons in enhancing effective template dosage in plant cells (Zhang and Mason, 2006).

From a practical point of view, our findings provide two key insights into the use of GVRs in plant systems. First, selecting the appropriate replicon backbone is crucial for different hosts. ToLCV and BeYDV GVRs consistently show strong potential for achieving sustained transient protein expression, particularly in the context of molecular farming or of the transient delivery of genome-editing reagents in tobacco plants. Second, although WDV GVR could serve as a viable alternative in tomato, since its performance in dicots may be most useful in applications where moderate but early expression is preferred. These conclusions support previous studies indicating that different GVR backbones vary in both expression efficiency and suitability for genome-editing applications across plant species (Dahan-Meir et al., 2018).

Finally, while “deconstructed” GVRs lack movement and coat protein genes (reducing concerns about systemic spread and germline transmission), biosafety considerations, off-target effects in genome editing, and the need to obtain replicon-free progeny (for heritable engineering) should guide future development. Coupling GVR delivery with transient expression systems or with inducible Rep expression could provide temporal control and mitigate potential risks. More importantly, translating these findings to genome-editing outcomes, such as measuring increases in targeted mutation or HDR frequencies, when editing cassettes are delivered on the same replicons, represents the logical next step for demonstrating practical gains in plant biotechnology workflows.

Overall, our results demonstrate that GVRs enhance and prolong transient gene expression in two solanaceous crops, with performance varying among replicon backbones and host species. ToLCV and BeYDV were most effective in tobacco, whereas ToLCV and WDV showed superior performance in tomato, highlighting their potential for transient protein production and for improving the efficiency of genome-editing reagents. With further mechanistic studies and optimization, these GVR platforms could be tailored to specific host-cargo combinations, expanding the toolkit for plant biotechnology and crop improvement.

## Data Availability

The raw data supporting the conclusions of this article will be made available by the authors, without undue reservation.
