# Supplementary material for: Geminivirus-derived replicons: assessment for transient GFP expression in tobacco and tomato
Source: Front Plant Sci. 2026 Jun 3;17:1857507. doi: 10.3389/fpls.2026.1857507 (PMC13272357; doi:10.3389/fpls.2026.1857507)
Supplement: Supplementary file 1 [file DataSheet1.docx]

**Geminivirus-derived Replicons: Assessment for Transient GFP Expression in Tobacco and Tomato.**

Nikolaos Tsakirpaloglou^1,2,*^, Ourania Melita^1,*^, Athanasios Kaldis^1^, Alexios Polidoros^2^, Andreas Voloudakis^1^.

^1^ Laboratory of Plant Breeding and Biometry, Faculty of Crop Science, Agricultural University of Athens, Athens 11855, Greece.

^2^ Laboratory of Genetics and Plant Breeding, Faculty of Agriculture, Aristotle University of Thessaloniki, 54124 Thessaloniki, Greece.

^*^ These authors contributed equally to the manuscript.

Correspondence: Andreas Voloudakis ([avoloud@aua.gr](mailto:avoloud@aua.gr))

**Supplementary Figure 1.** Multiple sequence alignment of the Long Intergenic Regions (LIRs) from the deconstructed geminivirus-derived replicons (GVRs) used in this study, performed using Clustal Omega.

| BeYDV_LIR      ---gagggtcgtacgaataattcgtatccaacggaaatacctgatacaatatacgctcca 57  ToLCV_LIR      ---------------------------------------------------tg--cccaa 7  WDV_LIR        cgagatgggctaccacgcacttccttata--agctaaggcatggcacagattt--cccc- 55                                                                    *   * *.  BeYDV_LIR      tcaaataccatcacatcgtatatgcttttatagtgtgaa-------cacctttaacccta 110  ToLCV_LIR      -----------------------g--tttaaata--gaaa-------------------- 20  WDV_LIR        -----------------------g--tcaaaagtgtgataaatcaacatcttccacgaaa 90                                        *  * :*:* :  **:  BeYDV_LIR      gtgggcgggaaattttctactttaaatctggacc----------gctcgtgc----taa- 155  ToLCV_LIR      --------------------------------------------acacaaaacaattaag 36  WDV_LIR        gtgatcagaaaa------------gatgtggtccaaaagcctccgcgcactcacgaaaag 138                                                             .* *.  .    :**  BeYDV_LIR      -agcactcgcgataagggggggccacgccggt-aatattaaattcggcgtgggccccccc 213  ToLCV_LIR      ctttgagcgcgtcaggtgattggtccgcatgtctttgtcagttattgcgttgtggggtcc 96  WDV_LIR        ccgagtgcgcgtcgggggccacc-acgcggggtaatattataacccgcgtggaggccccc 197                     .  ****: ..* *      .***  *  ::*.* * ::   **** *      **  BeYDV_LIR      ttgtcg------------------------------------------------------ 219  ToLCV_LIR      aaaaaa----aaaatcgcggccatccggtaatattatacggatggcc--gctt--tttgg 148  WDV_LIR        cgaccgcacaccgaacagggcccac------------acgattcgctacgctaccgtggg 245                   . ..  BeYDV_LIR      ----caaagacttcgtctttaagtaaattccacgtcattttc--cactatctattaaaat 273  ToLCV_LIR      agcgtaagggttttgattt-----------aaag--------ttccatatttacatttat 189  WDV_LIR        aacactagggccctgttct-----------cccgccaaaacctgccctatata-----aa 289                      :*.*.    *:  *           ...*          *..*** **     *:  BeYDV_LIR      gaccaa--------aatac----------ccctgcctccatgcctccacgccggttataa 315  ToLCV_LIR      gccattagagtctccata----tat---------ataggactccaatacaccgatacata 236  WDV_LIR        g-cattgg--acacattgcatttgcagtgtgcagaattcacacctccacgcagggtagag 346                 * *.::        .:*.                . :  *  **:. **.*.*. :. :.  BeYDV_LIR      gatagagtttgaggcaacccctcggagtcaca--a-------------caac-------- 352  ToLCV_LIR      gagagagttgagag----acaccgattgacca-------------agtcaac-------- 271  WDV_LIR        gatagagttttctggcacaccccgttttttcgctaaggacctgccggacttctgttcact 406                 ** ******    *    .*. ** :    *.                *::*  BeYDV_LIR      --- 352  ToLCV_LIR      --- 271  WDV_LIR        acc 409 |
| --- |

**Supplementary Figure 2.** Multiple sequence alignments of replication-associated proteins from the deconstructed GVRs used in this study, generated using Clustal Omega. The analyzed sequences include BeYDV (RepA: NCBI protein_id ABE67102.1; Rep: ABE67103.1), ToLCV (AC1: ABF71078.2; AC2: ABF71077.2; AC3: ABF71076.1; AC4: ABF71079.1), and WDV (Rep: QCZ25039.1; RepA: QCZ25038.1).

| **RepA_BeYDV vs WDV**  BeYDV_RepA      -MPSASKNFRLQSKYVFLTYPKCSSQRDDLFQFLWEKLTPFLIFFLGVASELHQDGTTHY 59  WDV_RepA        MASSSAPRFRVYSKYLFLTYPQCTLEPQYALDSLRTLLNKYEPLYIAAVRELHEDGSPHL 60                     *:: .**: ***:*****:*: : :  :: *   *. :  :::... ***:**: *  BeYDV_RepA      HALLQLDKKPCIRDPSFFD-------FEGNHPNIQPARNSKQVLDYISKDGDIKTR---- 108  WDV_RepA        HVLVQNKLRASITNPNALNLRMDTSPFSIFHPNIQAAKDCNQVRDYITKEVDSDVNTAEW 120                  *.*:* . : .* :*. ::       *.  ***** *::.:** ***:*: * ...  BeYDV_RepA      GDFRDHK-VSPRKSDARWRTIIQTATSKEEYLDMIKEEFPHEWATKLQWLEYSANKLFPP 167  WDV_RepA        GTFVAVSTPGRKDRDADMKQIIESSSSREEFLSMVCNRFPFEWSIRLKDFEYTARHLFPD 180                  * *   .  . :. **  : **::::*:**:*.*: :.**.**: :*: :**:*.:***  BeYDV_RepA      QPEPYVSPFTESDLRCHEDLHSWRETHLYHDNGRNGIRHPSLYICGPTRTG----KTTWA 223  WDV_RepA        PVATYTPEFPTESLICHETIESWKNEHLYSVSLES------YILCTSTPADQAQSDLEWM 234                      *.  *  ..* *** :.**:: ***  . ..        :*  * :.    .  *  BeYDV_RepA      RSLGRHNYWNGTIDFTNYDEHATYNIIDDIPFKFVPLWKQLIGCQSDFTVNPKYGKKKKI 283  WDV_RepA        DDYSRSH--RGGISPSTSAGQPE---QERLP-----------G----------------- 261                   . .* :  .* *. :.   :      : :*           *  BeYDV_RepA      KGGIPSIILCNPDEDWMLSMTSQQKDYFKDNCVTHYMCDGETFFARESSSH 334  WDV_RepA        -QGL----------------------------------------------- 264                    *:  **Rep_BeYDV vs WDV**  BeYDV_Rep      -MPSASKNFRLQSKYVFLTYPKCSSQRDDLFQFLWEKLTPFLIFFLGVASELHQDGTTHY 59  WDV_Rep        MASSSAPRFRVYSKYLFLTYPQCTLEPQYALDSLRTLLNKYEPLYIAAVRELHEDGSPHL 60                    *:: .**: ***:*****:*: : :  :: *   *. :  :::... ***:**: *  BeYDV_Rep      HALLQLDKKPCIRDPSFFD-------FEGNHPNIQPARNSKQVLDYISKDGDIKTR---- 108  WDV_Rep        HVLVQNKLRASITNPNALNLRMDTSPFSIFHPNIQAAKDCNQVRDYITKEVDSDVNTAEW 120                 *.*:* . : .* :*. ::       *.  ***** *::.:** ***:*: * ...  BeYDV_Rep      GDFRDHK-VSPRKSDARWRTIIQTATSKEEYLDMIKEEFPHEWATKLQWLEYSANKLFPP 167  WDV_Rep        GTFVAVSTPGRKDRDADMKQIIESSSSREEFLSMVCNRFPFEWSIRLKDFEYTARHLFPD 180                 * *   .  . :. **  : **::::*:**:*.*: :.**.**: :*: :**:*.:***  BeYDV_Rep      QPEPYVSPFTESDLRCHEDLHSWRETHLYHVSIDAYTYIHPVSYQQAQSDLEWMADLTRT 227  WDV_Rep        PVATYTPEFPTESLICHETIESWKNEHLYSESPGRHKSIYICGP-TRTGKTSWARSLGTH 239                     *.  *  ..* *** :.**:: ***  * . :. *:  .     .. .*  .*  BeYDV_Rep      M--EGMESDTPAS-TSADQLVPERPPGLEVS--------D-----------DTTIGTVPS 265  WDV_Rep        NYYNSLVDFTTYDVNAKYNIIDDIPFKFTPNWKCFVGAQRDFTVNPKYGKRKVIRGGIPC 299                    :.: . *  . .:  ::: : *  :  .                    ..  * :*.  BeYDV_Rep      ISPTTMNTPPIISSTTSPSSSSHCGSN--------------------------- 292  WDV_Rep        IILVNPDEDW-L-KDMTPEQSDYMYSNTVVHYMYEGETFINYSFASGEDVTASQ 351                 *  .. :    : .  :*..*.:  **  **BeYDV_Rep vs WDV_RepA**  BeYDV_Rep      -MPSASKNFRLQSKYVFLTYPKCSSQRDDLFQFLWEKLTPFLIFFLGVASELHQDGTTHY 59  WDV_RepA       MASSSAPRFRVYSKYLFLTYPQCTLEPQYALDSLRTLLNKYEPLYIAAVRELHEDGSPHL 60                    *:: .**: ***:*****:*: : :  :: *   *. :  :::... ***:**: *  BeYDV_Rep      HALLQLDKKPCIRDPSFFD-------FEGNHPNIQPARNSKQVLDYISKDGDIKTR---- 108  WDV_RepA       HVLVQNKLRASITNPNALNLRMDTSPFSIFHPNIQAAKDCNQVRDYITKEVDSDVNTAEW 120                 *.*:* . : .* :*. ::       *.  ***** *::.:** ***:*: * ...  BeYDV_Rep      GDFRDHKV-SPRKSDARWRTIIQTATSKEEYLDMIKEEFPHEWATKLQWLEYSANKLFPP 167  WDV_RepA       GTFVAVSTPGRKDRDADMKQIIESSSSREEFLSMVCNRFPFEWSIRLKDFEYTARHLFPD 180                 * *   .. . :. **  : **::::*:**:*.*: :.**.**: :*: :**:*.:***  BeYDV_Rep      QPEPYVSPFTESDLRCHEDLHSWRETHLYHVSIDAYTYIHPVSYQQAQSDLEWMADLTRT 227  WDV_RepA       PVATYTPEFPTESLICHETIESWKNEHLYSVSLESYILCTSTPADQAQSDLEWMDDYSRS 240                     *.  *  ..* *** :.**:: *** **:::*     .  :********* * :*:  BeYDV_Rep      MEGMESDTPASTSADQLVPERPPGLEVSDDTTIGTVPSISPTTMNTPPIISSTTSPSSSS 287  WDV_RepA       HRGGISP---STSAGQPEQERLPGQGL--------------------------------- 264                  .*  *    ****.*   ** **  :  BeYDV_Rep      HCGSN 292  WDV_RepA       ----- 264  **BeYDV_RepA vs WDV_Rep (~50% similarity)**  BeYDV_RepA      -MPSASKNFRLQSKYVFLTYPKCSSQRDDLFQFLWEKLTPFLIFFLGVASELHQDGTTHY 59  WDV_Rep         MASSSAPRFRVYSKYLFLTYPQCTLEPQYALDSLRTLLNKYEPLYIAAVRELHEDGSPHL 60                     *:: .**: ***:*****:*: : :  :: *   *. :  :::... ***:**: *  BeYDV_RepA      HALLQLDKKPCIRDPSFFD-------FEGNHPNIQPARNSKQVLDYISKDGDIKTR---- 108  WDV_Rep         HVLVQNKLRASITNPNALNLRMDTSPFSIFHPNIQAAKDCNQVRDYITKEVDSDVNTAEW 120                  *.*:* . : .* :*. ::       *.  ***** *::.:** ***:*: * ...  BeYDV_RepA      GDFRDHK-VSPRKSDARWRTIIQTATSKEEYLDMIKEEFPHEWATKLQWLEYSANKLFPP 167  WDV_Rep         GTFVAVSTPGRKDRDADMKQIIESSSSREEFLSMVCNRFPFEWSIRLKDFEYTARHLFPD 180                  * *   .  . :. **  : **::::*:**:*.*: :.**.**: :*: :**:*.:***  BeYDV_RepA      QPEPYVSPFTESDLRCHEDLHSWRETHLYHDNGRNGIRHPSLYICGPTRTGKTTWARSLG 227  WDV_Rep         PVATYTPEFPTESLICHETIESWKNEHLYSESP---GRHKSIYICGPTRTGKTSWARSLG 237                      *.  *  ..* *** :.**:: *** :.     ** *:***********:******  BeYDV_RepA      RHNYWNGTIDFTNYDEHATYNIIDDIPFKFVPLWKQLIGCQSDFTVNPKYGKKKKIKGGI 287  WDV_Rep         THNYYNSLVDFTTYDVNAKYNIIDDIPFKFTPNWKCFVGAQRDFTVNPKYGKRKVIRGGI 297                   ***:*. :***.** :*.***********.* ** ::*.* **********:* *:***  BeYDV_RepA      PSIILCNPDEDWMLSMTSQQKDYFKDNCVTHYMCDGETFFARESSSH------- 334  WDV_Rep         PCIILVNPDEDWLKDMTPEQSDYMYSNTVVHYMYEGETFINYSFASGEDVTASQ 351                  *.*** ******: .** :*.**: .* *.*** :****:  . :*  **BeYDV_RepA vs ToLCV-AC1**  BeYDV_RepA      MPSASKNFRLQSKYVFLTYPKCSSQRDDLFQFLWEKLTPFLIFFLGVASELHQDGTTHYH 60  ToLCV-AC1       -MAAPNRFKINAKNYFLTYPKCSLTKEEHFPKLLNLQTPTFKKFIRICRELHEDGTPHLH 59                    :* :.*::::*  ********  ::: *  * :  ** :  *: :. ***:*** * *  BeYDV_RepA      ALLQLDKKPCIRDPSFFDFEG------NHPNIQPARNSKQVLDYISKDGDIKTRGDFRDH 114  ToLCV-AC1       VLIQFEGKFQCKNNRFFDLTSPTRSAHFHPNIQGAKSSSDVKSYMEKDGDVIDHGVFQVD 119                  .*:*:: *   ::  ***: .       ***** *:.*.:* .*:.****:  :* *: .  BeYDV_RepA      KVSPRKSDA----RWRTIIQTATSKEEYLDMIKEEFPHEWATKLQWLEYSANKLFPPQPE 170  ToLCV-AC1       GRSARGGCQSANDAYAEAI-NSGSKAQALNILREKAPKDFVLQFHNLNSNLDRIFTPPMD 178                    * * .       :   * .: ** : *::::*: *:::. ::: *: . :::* *  :  BeYDV_RepA      PYVSPFTESDLR-CHEDLHSWRETHLYHDNGRNGIRHPSLYICGPTRTGKTTWARSLGRH 229  ToLCV-AC1       VYVSPFLSSSFDQVPEELEEWAAENVCSPA-ARPLRPISIVIEGDSRTGKTMWARSLGPH 237                   ***** .*.:    *:*..*   ::      . :*  *: * * :***** ****** *  BeYDV_RepA      NYWNGTIDFT--NYDEHATYNIIDDIPFKFVPLWKQLIGCQSDFTVNPKYGKKKKIKGGI 287  ToLCV-AC1       NYLCGHLDLSPKVYSNDAWYNVIDDVDPHYLKHFKEFMGAQRDWQSNTKYGKPVQIKGGI 297                  **  * :*::   *.:.* **:***:  :::  :*:::*.* *:  * ****  :*****  BeYDV_RepA      PSIILCNPDEDWMLSMTSQQKDYFKDNCVTHYMCDGETFFARESSSH------------- 334  ToLCV-AC1       PTTFLCNPGPNSSYKEYLDE---EKNSALKNWALKNATFVTLEGPLYSGTNQSTAQASQE 354                  *: :****. :   .   ::    *:..:.::  .. **.: *.  :  BeYDV_RepA      ------- 334  ToLCV-AC1       GDQTSTS 361  **BeYDV_RepA vs ToLCV-AC2**  BeYDV_RepA      MPSASKNFRLQSKYVFLTYPKCSSQRDDLFQFLWEKLTPFLIFFLGVASELHQDGTTHYH 60  ToLCV-AC2       ------------------------------------------------------------ 0    BeYDV_RepA      ALLQLDKKPCIRDPSFFDFEGNHPNIQPARNSKQ--------VLDYISKDGDIKTRGDFR 112  ToLCV-AC2       -------------------------MRPSSPSKAHSTQVPIKVQHRLAKKGTRRRRVDLP 35                                           ::*:  **         * . ::*.*  : * *:  BeYDV_RepA      DHKVSPRKSDARWRTIIQTATSKEEYLDMIKEEFPHEWATKLQWLEYSANK---LFPPQP 169  ToLCV-AC2       ------C--GCSY--FIAITCHDHGF----THRGTHHCSSSREWRVYLGDSKSPIFQDNR 81                           .. :  :*  :  .. :    ...  *. ::. :*  * .:.   :*  :  BeYDV_RepA      EPYVSPFTESDLRCHEDLHSWRETHLYHDNGRNGIRHPSLYICGPTRTGKTTWARSLGRH 229  ToLCV-AC2       APK--PALRDERRHH--------------------HRPDTVQSQP--------EESVGDT 111                   *   *  ..: * *                    ::*.   . *         .*:*  BeYDV_RepA      NYWNGTIDFTNYDEHATYNIIDDIPFKFVPLWKQLIGCQSDFTVNPKYGKKKKIKGGIPS 289  ToLCV-AC2       QMFPNLP------------NLDD---LTASDWSFLKGI---------------------- 134                  : : .               :**     .  *. * *  BeYDV_RepA      IILCNPDEDWMLSMTSQQKDYFKDNCVTHYMCDGETFFARESSSH 334  ToLCV-AC2       --------------------------------------------- 134  **BeYDV_RepA vs ToLCV-AC3**  BeYDV_RepA      MPSASKNFRLQSKYVFLTYPKCSSQRDDLFQFLWEKLTPFLIFFLGVASELH---QDG-- 55  ToLCV-AC3       MDSRTGE-------------LITAAQAENGVFIWEIQNPLYFKITEHQNRPFVMNEDIIT 47                  * * : :               :: : :   *:**  .*: : :    .. .   :*  BeYDV_RepA      --TTHYHAL-LQLDKKPCIRDPSFFDFEGNHPNI-QPARNSKQVLDYISKDGDIKTRGDF 111  ToLCV-AC3       VRIQFNHNLRKVLGIHKCFLTYRIWMTS-QPPTGRFLRVFKTQVLKFLNNLGVISLNNVI 106                      . * *   *. : *:    ::  . : *.       ..***.::.: * *. .. :  BeYDV_RepA      RDHKVSPRKSDARWRTIIQTATSKEEYLDMIKEEFPHEWATKLQWLEYSANKLFPPQPEP 171  ToLCV-AC3       R------AVNYVLWDALTQTTFVD------------------------------------ 124                  *        . . * :: **:  .  BeYDV_RepA      YVSPFTESDLRCHEDLHSWRETHLYHDNGRNGIRHPSLYICGPTRTGKTTWARSLGRHNY 231  ToLCV-AC3       ------------------------------------------------------------ 124    BeYDV_RepA      WNGTIDFTNYDEHATYNIIDDIPFKFVPLWKQLIGCQSDFTVNPKYGKKKKIKGGIPSII 291  ToLCV-AC3       ---------SSHIIKFNIY----------------------------------------- 134                            ..  .:**  BeYDV_RepA      LCNPDEDWMLSMTSQQKDYFKDNCVTHYMCDGETFFARESSSH 334  ToLCV-AC3       ------------------------------------------- 134  **BeYDV_RepA vs ToLCV-AC4**  BeYDV_RepA      MPSASKNFRLQSKYVFLTYPKCSSQRDDLFQFLWEKLTPFLIFFLGVASELHQDGTTHYH 60  ToLCV-AC4       --------------------------------------------MG-------------- 2                                                              :*  BeYDV_RepA      ALLQLDKKPCIRDPSF--------FDFEGNHPNIQPARNSKQVLDYISKDGDIKTRGDFR 112  ToLCV-AC4       ------LLTCMCSSSSKESSSAKTIDSSTSHP--QP--------------GQHISIRTFR 40                           *: . *         :* . .**  **              *:  :   **  BeYDV_RepA      DHKVSPRKSDARWRTIIQTATSKEEYLDMIKEEFPHEWATKLQWLEYSANKLFPPQPEPY 172  ToLCV-AC4       ELRA-QAMSNPTWKKTETSL----------IMEFSKSMDDQLE----------------- 72                  : :.    *:  *:.   :             ** :.   :*:  BeYDV_RepA      VSPFTESDLRCHEDLHSWRETHLYHDNGRNGIRHPSLYICGPTRTGKTTWARSLGRHNYW 232  ToLCV-AC4       -------------EVANLPTTHMPRQSIQGPKLRPLIY---------------------- 97                               :: .   **: ::. :.   :* :*  BeYDV_RepA      NGTIDFTNYDEHATYNIIDDIPFKFVPLWKQLIGCQSDFTVNPKYGKKKKIKGGIPSIIL 292  ToLCV-AC4       ------------------------------------------------------------ 97    BeYDV_RepA      CNPDEDWMLSMTSQQKDYFKDNCVTHYMCDGETFFARESSSH 334  ToLCV-AC4       ------------------------------------------ 97  **BeYDV_Rep vs ToLCV-AC1 (~50% similarity)**  BeYDV_Rep      MPSASKNFRLQSKYVFLTYPKCSSQRDDLFQFLWEKLTPFLIFFLGVASELHQDGTTHYH 60  ToLCV-AC1      -MAAPNRFKINAKNYFLTYPKCSLTKEEHFPKLLNLQTPTFKKFIRICRELHEDGTPHLH 59                   :* :.*::::*  ********  ::: *  * :  ** :  *: :. ***:*** * *  BeYDV_Rep      ALLQLDKKPCIRDPSFFDFEG------NHPNIQPARNSKQVLDYISKDGDIKTRGDFRDH 114  ToLCV-AC1      VLIQFEGKFQCKNNRFFDLTSPTRSAHFHPNIQGAKSSSDVKSYMEKDGDVIDHGVFQVD 119                 .*:*:: *   ::  ***: .       ***** *:.*.:* .*:.****:  :* *: .  BeYDV_Rep      KVSPRKSDA----RWRTIIQTATSKEEYLDMIKEEFPHEWATKLQWLEYSANKLFPPQPE 170  ToLCV-AC1      GRSARGGCQSANDAYAEAI-NSGSKAQALNILREKAPKDFVLQFHNLNSNLDRIFTPPMD 178                   * * .       :   * .: ** : *::::*: *:::. ::: *: . :::* *  :  BeYDV_Rep      PYVSPFTESDLR-CHEDLHSWRETHLYHVSIDAYTYIHPVSYQ-QAQSDLEWMADLTRT- 227  ToLCV-AC1      VYVSPFLSSSFDQVPEELEEWAAENVCSPAARPLRPISIVIEGDSRTGKTMWARSLGPHN 238                  ***** .*.:    *:*..*   ::   :      *  *    .  ..  *  .*  BeYDV_Rep      -MEGMESDTPASTSADQ---LVPERPPGLE------------VSDDTTIGTVPSI---SP 268  ToLCV-AC1      YLCGHLDLSPKVYSNDAWYNVIDDVDPHYLKHFKEFMGAQRDWQSNTKYGKPVQIKGGIP 298                  : *  . :*   * *    :: :  *                ..:*. *.  .*    *  BeYDV_Rep      TTMN-T---------------------------------PPIISSTT-SPSSSSHCGSN- 292  ToLCV-AC1      TTFLCNPGPNSSYKEYLDEEKNSALKNWALKNATFVTLEGPLYSGTNQSTAQASQEGDQT 358                 **:  .                                  *: *.*. * :.:*: *.:  BeYDV_Rep      --- 292  ToLCV-AC1      STS 361  **BeYDV_Rep vs ToLCV-AC2**  BeYDV_Rep      MPSASKNFRLQSKYVFLTYPKCSSQRDDLFQFLWEKLTPFLIFFLGVASELHQDGTTHYH 60  ToLCV-AC2      ------------------------------------------------------------ 0    BeYDV_Rep      ALLQLDKKPCIRDPSFFDFEGNHPNIQPARNSKQ--------VLDYISKDGDIKTRGDFR 112  ToLCV-AC2      -------------------------MRPSSPSKAHSTQVPIKVQHRLAKKGTRRRRVDL- 34                                          ::*:  **         * . ::*.*  : * *:  BeYDV_Rep      DHKVSPRKSDARWRTIIQTATSKEEYLDMIKEEFPHEWATKLQWLEYSANKLFPPQPEPY 172  ToLCV-AC2      ------------------------------------------------------PCGCSY 40                                                                       *    *  BeYDV_Rep      VSPFTESDLRCHEDLHSWRETHLYHVSIDAYTYIHPVSYQQAQSDLEWMADLTRTMEGME 232  ToLCV-AC2      F-----IAITCHDHGFTHRGTHH------------------CSSSREWRVYLGDSKSPIF 77                 .       : **:. .: * **                   ..*. ** . *  : . :  BeYDV_Rep      SDTPA--STSAD-QLVPERPPGL------EVSDDTTIGTVPSISPTTMNTPPIISSTTSP 283  ToLCV-AC2      QDNRAPKPALRDERRHHHRPDTVQSQPEESVGDTQMFPNLPNLDDLTASDWSFLKGI--- 134                 .*. *   :  * :   .**  :      .*.*   : .:*.:.  * .   ::..  BeYDV_Rep      SSSSHCGSN 292  ToLCV-AC2      --------- 134  **BeYDV_Rep vs ToLCV-AC3**  BeYDV_Rep      MPSASKNFRLQSKYVFLTYPKCSSQRDDLFQFLWEKLTPFLIFFLGVASELH---QDG-- 55  ToLCV-AC3      MDSRTGE-------------LITAAQAENGVFIWEIQNPLYFKITEHQNRPFVMNEDIIT 47                 * * : :               :: : :   *:**  .*: : :    .. .   :*  BeYDV_Rep      --TTHYHAL-LQLDKKPCIRDPSFFDFEGNHPNI-QPARNSKQVLDYISKDGDIKTRGDF 111  ToLCV-AC3      VRIQFNHNLRKVLGIHKCFLTYRIWMTS-QPPTGRFLRVFKTQVLKFLNNLGVISLNNVI 106                     . * *   *. : *:    ::  . : *.       ..***.::.: * *. .. :  BeYDV_Rep      RDHKVSPRKSDARWRTIIQTATSKEEYLDMIKEEFPHEWATKLQWLEYSANKLFPPQPEP 171  ToLCV-AC3      R------AVNYVLWDALTQTTFVDSS---------------------------------- 126                 *        . . * :: **:  ...  BeYDV_Rep      YVSPFTESDLRCHEDLHSWRETHLYHVSIDAYTYIHPVSYQQAQSDLEWMADLTRTMEGM 231  ToLCV-AC3      ----------------------HIIK--FNIY---------------------------- 134                                       *: :  :: *  BeYDV_Rep      ESDTPASTSADQLVPERPPGLEVSDDTTIGTVPSISPTTMNTPPIISSTTSPSSSSHCGS 291  ToLCV-AC3      ------------------------------------------------------------ 134    BeYDV_Rep      N 292  ToLCV-AC3      - 134  **BeYDV_Rep vs ToLCV-AC4**  BeYDV_Rep      MPSASKNFRLQSKYVFLTYPKCSSQRDDLFQFLWEKLTPFLIFFLGVASELHQDGTTHYH 60  ToLCV-AC4      ------------------------------------------------------------ 0    BeYDV_Rep      ALLQLDKKPCIRDPSF--------FDFEGNHPNIQPARNSKQVLDYISKDGDIKTRGDFR 112  ToLCV-AC4      ----MGLLTCMCSSSSKESSSAKTIDSSTSHP--QP--------------GQHISIRTFR 40                     :.   *: . *         :* . .**  **              *:  :   **  BeYDV_Rep      DHKVSPRKSDARWRTIIQTATSKEEYLDMIKEEFPHEWATKLQWLEYSANKLFPPQPEPY 172  ToLCV-AC4      ELRA-QAMSNPTWKKTETSL----------IMEFSKSMDDQLE----------------- 72                 : :.    *:  *:.   :             ** :.   :*:  BeYDV_Rep      VSPFTESDLRCHEDLHSWRETHLYHVSIDAYTYIHPVSYQQAQSDLEWMADLTRTMEGME 232  ToLCV-AC4      -------------EVANLPTTHMPRQSIQG-PKLRPLIY--------------------- 97                              :: .   **: : **:.   ::*: *  BeYDV_Rep      SDTPASTSADQLVPERPPGLEVSDDTTIGTVPSISPTTMNTPPIISSTTSPSSSSHCGSN 292  ToLCV-AC4      ------------------------------------------------------------ 97    **WDV_Rep vs ToLCV-AC1**  WDV_Rep        MASSSAPRFRVYSKYLFLTYPQCTLEPQYALDSLRTLLNKYEPLYIAAVRELHEDGSPHL 60  ToLCV-AC1      --MAAPNRFKINAKNYFLTYPKCSLTKEEHFPKLLNLQTPTFKKFIRICRELHEDGTPHL 58                    ::  **:: :*  *****:*:*  :  : .* .* .     :*   *******:***  WDV_Rep        HVLVQNKLRASITNPNALNLRMDTSPFSIFHPNIQAAKDCNQVRDYITKEVDSDVNTAEW 120  ToLCV-AC1      HVLIQFEGKFQCKNNRFFDLTS-PTRSAHFHPNIQGAKSSSDVKSYMEKDGDV----IDH 113                 ***:* : : . .* . ::*    :  : ******.**...:*:.*: *: *      :  WDV_Rep        GTFVAVSTPGRKD--RDADMKQIIESSSSREEFLSMVCNRFPFEWSIRLKDFEYTARHLF 178  ToLCV-AC1      GVFQVDGRSARGGCQSANDAYAEAINSGSKAQALNILREKAPKDFVLQFHNLNSNLDRIF 173                 *.* . .  .* .     *      .*.*: : *.:: :: * :: ::::::: .  ::*  WDV_Rep        PDPVATYTPEFPTESL-ICHETIESWKNEHLYSE--SPGRHKSIYICGPTRTGKTSWARS 235  ToLCV-AC1      TPPMDVYVSPFLSSSFDQVPEELEEWAAENVCSPAARPLRPISIVIEGDSRTGKTMWARS 233                   *: .*.  * :.*:    * :*.*  *:: *    * *  ** * * :***** ****  WDV_Rep        LGTHNYYNSLVDFT--TYDVNAKYNIIDDIPFKFTPNWKCFVGAQRDFTVNPKYGKRKVI 293  ToLCV-AC1      LGPHNYLCGHLDLSPKVYSNDAWYNVIDDVDPHYLKHFKEFMGAQRDWQSNTKYGKPVQI 293                 ** ***  . :*::  .*. :* **:***:  ::  ::* *:*****:  * ****   *  WDV_Rep        RGGIPCIILVNPDEDWLKD-MTPEQSDYMYSNTVVHYMYEGETFINYS--FASGEDVTAS 350  ToLCV-AC1      KGGIPTTFLCNPGPNSSYKEYLDEEKN----SALKNWALKNATFVTLEGPLYSGTNQSTA 349                 :****  :* **. :   .    *:.:    .:: ::  :. **:. .  : ** : :::  WDV_Rep        Q----------- 351  ToLCV-AC1      QASQEGDQTSTS 361                 *  **WDV_Rep vs ToLCV-AC2**  WDV_Rep        MASSSAPRFRVYSKYLFLTYPQCTLEPQYALDSLRTLLNKYEPLYIAAVRELHEDGSPHL 60  ToLCV-AC2      ------------------------MRPSS-------------P---------SKAHSTQV 14                                         :.*.              *          :  * ::  WDV_Rep        HVLVQNKLRASITNPNALNLRMDTSPFSIFHPNIQAAKDCNQVRDYITKEVDSDVNTAEW 120  ToLCV-AC2      PIKVQHRLAKK------------------------------------------------- 25                  : **::*  .  WDV_Rep        GTFVAVSTPGRKDRDADMKQIIESSSSREEFLSMVCNRFPF------------EWSIRLK 168  ToLCV-AC2      ---------GTR------RRRVDLPCGCSYFIAITCHDHGFTHRGTHHCSSSREWRVYLG 70                          * :      :: ::  .. . *:::.*: . *            ** : *  WDV_Rep        DFEYTARHLFPDPVATYTPEFPTESLICHETIESWKNEHLYSESPGRHKSIYICGPTRTG 228  ToLCV-AC2      DSKS---PIFQDNRAP-KPALRDERRH--------------HHRPD------------TV 100                 * :     :* *  *  .* :  *                  . *.            *  WDV_Rep        KTSWARSLGTHNYYNSLVDFTTYDVNAKYNIIDDIPFKFTPNWKCFVGAQRDFTVNPKYG 288  ToLCV-AC2      QSQPEESVGDTQMFPNLPNL---------------DDLTASDWSFLKGI----------- 134                 ::.  .*:*  : : .* ::                   : :*. : *  WDV_Rep        KRKVIRGGIPCIILVNPDEDWLKDMTPEQSDYMYSNTVVHYMYEGETFINYSFASGEDVT 348  ToLCV-AC2      ------------------------------------------------------------ 134    WDV_Rep        ASQ 351  ToLCV-AC2      --- 134  **WDV_Rep vs ToLCV-AC3**  WDV_Rep        MASSSAPRFRVYSKYLFLTYPQCTLEPQYALDSLRTLLNKYEPLYIAAVRELHEDGSPHL 60  ToLCV-AC3      ------------------------------------------------------------ 0    WDV_Rep        HVLVQNKLRASITNPNALNLRMDTSPFSIFHPNIQAAKDCNQVRDYITKEVDSDVNTAEW 120  ToLCV-AC3      ------------------------------------------------------------ 0    WDV_Rep        GTFVAVSTPGRKDRDADMKQIIESSSSREEFLSMVCNRFPFEWSIRLKDFEYTARHL--- 177  ToLCV-AC3      -------------MDSRTGELIT---------AAQAENGVFIWEIQNPLYFKITEHQNRP 38                               *:   ::*          :  .:.  * *.*:   :   :.*  WDV_Rep        --FPDPVATYTPEFPT------ESLICHETIESWKNEHLYSESPGRHKSIYICGPTRTGK 229  ToLCV-AC3      FVMNEDIITVRIQFNHNLRKVLGIHKCFLTYRIWMTSQ---PPTGRFLRVFK-----TQV 90                   : : : *   :*            *. * . * ..:      **.  ::      *  WDV_Rep        TSWARSLGTHNYYNS--LVDFTTYDVNAKYNIIDDIPFKFTPNWKCFVGAQRDFTVNPKY 287  ToLCV-AC3      LKFLNNLGVISLNNVIRAVNYVLWDALTQ------------------------------- 119                  .: ..**. .  *    *::. :*. ::  WDV_Rep        GKRKVIRGGIPCIILVNPDEDWLKDMTPEQSDYMYSNTVVHYMYEGETFINYSFASGEDV 347  ToLCV-AC3      ------------------------------TTFVDSSHII----------KFNIY----- 134                                               : :: *. ::          ::.:  WDV_Rep        TASQ 351  ToLCV-AC3      ---- 134  **WDV_Rep vs ToLCV-AC4**  WDV_Rep        MASSSAPRFRVYSKYLFLTYPQCTLEPQYALDSLRTLLNKYEPLYIAAVRELHEDGSPHL 60  ToLCV-AC4      ------------------------------------------------------------ 0    WDV_Rep        HVLVQNKLRASITNPNALNLRMDTSPFSIFHPNIQAAKDCNQVRDYITKEVDSDVNTAEW 120  ToLCV-AC4      -----------------------------------------------------------M 1    WDV_Rep        GTFVAVSTPGRKDRDADMKQIIESSSSREEFLSMVCNRFPFEWSIRLKDFEYTARHLFPD 180  ToLCV-AC4      GL-----------------------------LTCMCSSSSKESS-S-------------- 17                 *                              *: :*.    * *  WDV_Rep        PVATYTPEFPTESLICHETIESWKNEHLYSESPGRHKSIYICGPTR---TGKTSWARSLG 237  ToLCV-AC4      ----------------AKTID----SSTSHPQPGQHISIRTFRELRAQAMSNPTWKKTET 57                                  :**:    .     .**:* **      *    .: :* ::  WDV_Rep        THNYYNSLVDFTTYDVNAKYNIIDDIPFKFTPNWKCFVGAQRDFTVNPKYGKRKVIRGGI 297  ToLCV-AC4      S-----LIMEFSK-SMDDQLEEVANLPTTHMPR--------------------QSIQGP- 90                 :      :::*:. .:: : : : ::* .. *.                    : *:*  WDV_Rep        PCIILVNPDEDWLKDMTPEQSDYMYSNTVVHYMYEGETFINYSFASGEDVTASQ 351  ToLCV-AC4      ------------------KLRPLIY----------------------------- 97                                   :    :*  **WDV_RepA vs ToLCV-AC1**  WDV_RepA       MASSSAPRFRVYSKYLFLTYPQCTLEPQYALDSLRTLLNKYEPLYIAAVRELHEDGSPHL 60  ToLCV-AC1      --MAAPNRFKINAKNYFLTYPKCSLTKEEHFPKLLNLQTPTFKKFIRICRELHEDGTPHL 58                    ::  **:: :*  *****:*:*  :  : .* .* .     :*   *******:***  WDV_RepA       HVLVQNKLRASITNPNALNLRMDTSPFSIFHPNIQAAKDCNQVRDYITKEVDSDVNTAEW 120  ToLCV-AC1      HVLIQFEGKFQCKNNRFFDLTS-PTRSAHFHPNIQGAKSSSDVKSYMEKDGDV----IDH 113                 ***:* : : . .* . ::*    :  : ******.**...:*:.*: *: *      :  WDV_RepA       GTFVAVSTPGRKD---RDADMKQIIESSSSREEFLSMVCNRFPFEWSIRLKDFEYTARHL 177  ToLCV-AC1      GVFQVDGRSARGGCQSANDAYAEAI-NSGSKAQALNILREKAPKDFVLQFHNLNSNLDRI 172                 *.* . .  .* .    :    : * .*.*: : *.:: :: * :: ::::::: .  ::  WDV_RepA       FPDPVATYTPEFPTESL-ICHETIESWKNEHLYSVSLESYILCT-STPADQAQSDLEWMD 235  ToLCV-AC1      FTPPMDVYVSPFLSSSFDQVPEELEEWAAENVCSPAARPLRPISIVIEGDSRTGKTMWAR 232                 *  *: .*.  * :.*:    * :*.*  *:: * : .     :    .*.  ..  *  WDV_RepA       DYSRSH--RG--GIS-----------------------------------PSTSAGQPEQ 256  ToLCV-AC1      SLGPHNYLCGHLDLSPKVYSNDAWYNVIDDVDPHYLKHFKEFMGAQRDWQSNTKYGKPVQ 292                 . .  :   *  .:*                                    .*. *:* *  WDV_RepA       ER--L-------PGQGL------------------------------------------- 264  ToLCV-AC1      IKGGIPTTFLCNPGPNSSYKEYLDEEKNSALKNWALKNATFVTLEGPLYSGTNQSTAQAS 352                  :  :       ** .  WDV_RepA       --------- 264  ToLCV-AC1      QEGDQTSTS 361    **WDV_RepA vs ToLCV-AC2**  WDV_RepA       MASSSAPRFRVYSKYLFLTYPQCTLEPQYALDSLRTLLNKYEPLYIAAVRELHEDGSPHL 60  ToLCV-AC2      ------------------------MRPSS-------------P---------SKAHSTQV 14                                         :.*.              *          :  * ::  WDV_RepA       HVLVQNKLRASITNPNALNLRMDTSPFSIFHPNIQAAKDCNQVRDYITKEVDSDVNTAEW 120  ToLCV-AC2      PIKVQHRLAKKGTRRRRVDLPCGCSYFIA--------ITCHD-HGFTHRGTHHCSSSREW 65                  : **::*  . *. . ::*  . * *            *:: :.:  : ..   .: **  WDV_RepA       GTFVAVSTPGRKDRDADMKQIIESSSSREEFLSMVCNRFPFEWSIRLKDFEYTARHLFPD 180  ToLCV-AC2      RVYLGDSKSPIFQDNRAPKPALRD---------------------------ERRHHHRPD 98                  .::. *.    : :   *  :..                              :*  **  WDV_RepA       PVAT-------YTPEFPTESLICHETIESWKNEHLYSVSLESYILCTSTPADQAQSDLEW 233  ToLCV-AC2      TVQSQPEESVGDTQMFPNLPNLDDLTASDWS----------------------------F 130                  * :        *  **.   : . * ..*.                            :  WDV_RepA       MDDYSRSHRGGISPSTSAGQPEQERLPGQGL 264  ToLCV-AC2      LK--------GI------------------- 134                 :.        **  **WDV_RepA vs ToLCV-AC3**  WDV_RepA       MASSSAPRFRVYSKYLFLTYPQCTLEPQYALDSLRTLLNKYEPLYIAAVRELHEDGSPHL 60  ToLCV-AC3      MDSRTGELI-----------------TAAQAENGVFIWEIQNPLYFKITE---------- 33                 * * :.  :                      :.   : :  :***:  ..  WDV_RepA       HVLVQNKLRASITNPNALNLRMDTSPFSIFHPNIQAAKDCNQ-VRDYI------------ 107  ToLCV-AC3      -----HQNRPFVMNEDIITVRIQF---NHNLRKVLGIHKCFLTYRIWMTSQPPTGRFLRV 85                      :: *  : * : :.:*::    .    :: . :.*    * ::  WDV_RepA       ----------------TKEVDSDVNTAEWGTFVAVSTPGRKDRDADMKQIIESSSSREEF 151  ToLCV-AC3      FKTQVLKFLNNLGVISLNNVIRAVNYVLWDALTQTTF----------------------- 122                                  ::*   ** . *.::. .:  WDV_RepA       LSMVCNRFPFEWSIRLKDFEYTARHLFPDPVATYTPEFPTESLICHETIESWKNEHLYSV 211  ToLCV-AC3      ---------------------------VD-----------S-------------SHII-- 129                                             *           .             .*:  WDV_RepA       SLESYILCTSTPADQAQSDLEWMDDYSRSHRGGISPSTSAGQPEQERLPGQGL 264  ToLCV-AC3      KFNIY------------------------------------------------ 134                 .:: *  **WDV_RepA vs ToLCV-AC4**  WDV_RepA       MASSSAPRFRVYSKYLFLTYPQCTLEPQYALDSLRTLLNKYEPLYIAAVRELHEDGSPHL 60  ToLCV-AC4      -----------------MGLLTCMCSSSS------------------------------- 12                                  :    *  . .  WDV_RepA       HVLVQNKLRASITNPNALNLRMDTSPFSIFHPNIQAAKDCNQVRDYITKEVDSDVNTAEW 120  ToLCV-AC4      ------------------------------------------KESSSAKTIDSSTSHPQP 30                                                            ..  :* :**...  :  WDV_RepA       GTFVAVSTPGRKDRDADMKQIIESSSSREEFLSMVCNRFPFEWSIRLKDFEYTARHLFPD 180  ToLCV-AC4      GQHIS-----------------------------------------IRTFRELRAQAM-- 47                 * .::                                         :: *.    : :  WDV_RepA       PVATYTPEFPTESLICHETIESWKNEHLYSVSLESYILCTSTPADQAQSDLEWMDDYSRS 240  ToLCV-AC4      ------------------SNPTWKKTET-----------------------SLIMEFSKS 66                                   :  :**: .                        . : ::*:*  WDV_RepA       HRGGISPSTSAGQPEQERLPGQGL---------- 264  ToLCV-AC4      MDDQLEEVA---NLPTTHMPRQSIQGPKLRPLIY 97                   . :.  :   :    ::* *.: |
| --- |

**Supplementary Figure 3.** Green and red fluorescence channels of tobacco leaves (6 dpi) and tomato cotyledons (7 dpi) expressing the ToLCV replicon, visualized using ImageJ. Randomly selected rectangular regions of interest (visible in the green channel) are outlined and labeled with their corresponding identification numbers.

|  |
| --- |
